# Validation of Controlled Attenuation Parameter Measured by FibroScan as a Novel Surrogate Marker for the Evaluation of Metabolic Derangement

**DOI:** 10.3389/fendo.2021.739875

**Published:** 2022-01-31

**Authors:** Zhimin Huang, Kaka Ng, Hongyan Chen, Wanping Deng, Yanbing Li

**Affiliations:** ^1^ Department of Endocrinology and Diabetes Center, The First Affiliated Hospital of Sun Yat-sen University, Guangzhou, China; ^2^ Centro Hospitalar Conde de S Januário, Macau, Macau SAR, China; ^3^ Department of Endocrinology, Panyu District Traditional Chinese Medicine Hospital, Guangzhou, China

**Keywords:** non-alcoholic fatty liver disease (NAFLD), controlled attenuation parameter (CAP), metabolic syndrome (MetS), diabetes mellitus, insulin resistance, FibroScan

## Abstract

**Background/Objectives:**

Renaming non-alcoholic fatty liver disease (NAFLD) to metabolic dysfunction-associated fatty liver disease (MAFLD) suggests a shift of emphasis to the accompanying metabolic disturbance. Controlled attenuation parameter (CAP) measured by FibroScan has been shown to be correlated with hepatic steatosis. We aim to validate its usefulness as a novel surrogate marker for evaluating metabolic derangement.

**Subjects/Methods:**

Volunteers were recruited from medical staff at our hospital to undergo CAP measurements. Anthropometrics, CAP, and laboratory assessments for metabolic profiles and insulin resistance were collected. CAP < 238 dB/m denoted no hepatic steatosis, 238 ≤ CAP ≤ 259 dB/m denoted mild, 260 ≤ CAP ≤ 291 dB/m denoted moderate, and CAP > 291 dB/m denoted severe hepatic steatosis according to previous reports.

**Results:**

Data of 824 participants were included for analysis. The age was 53.2 ± 15.4 years, body mass index (BMI) was 23.6 ± 3.1 kg/m^2^, 24.4% were male subjects, and 22.0% met the criteria for metabolic syndrome (MetS). Taking the group with CAP < 238 dB/m as control, subjects with mild, moderate, and severe hepatic steatosis had increased odds of MetS by 3.51-, 3.32-, and 5.12-fold, respectively, after adjusting for multiple confounders (*p* = 0.020). Metabolic profiles, insulin resistance, and presence of MetS were similar between normal-weight subjects with CAP ≥ 238 dB/m and overweight subjects with CAP < 238 dB/m. Even in subjects with no MetS components, those with CAP ≥ 238 dB/m had higher BMI, waist circumferences, uric acid, triglyceride, white blood cell count, and insulin resistance, whereas lower adiponectin and estimated glomerular filtration rate. Waist circumference [OR 1.11 (1.04, 1.18), *p* = 0.001] and homeostatic model assessment of insulin resistance (HOMA-IR) [OR 2.39 (1.18, 4.83), *p* = 0.016] were predictive of hepatic steatosis according to CAP ≥ 238 dB/m.

**Conclusions:**

CAP is a convenient, sensitive, and non-invasive indicator for metabolic derangement. Prospective studies are needed to further validate its usefulness as a surrogate marker for the transition of metabolic status over time.

## Introduction

Non-alcoholic fatty liver disease (NAFLD) affects nearly one billion people worldwide and is expected to be the leading cause of end-stage liver disease in the coming decades (1). Increasing evidence has indicated a link between NAFLD and metabolic dysfunction and subsequent cardiovascular and renal complications. Due to accumulated understanding of the pathogenesis and prevalence of NAFLD and its comorbidities, including obesity and diabetes mellitus, the nomenclature and diagnostic criteria have recently been revised by a panel of international experts from 22 countries (2). Metabolic dysfunction-associated fatty liver disease (MAFLD) has been proposed to replace NAFLD. Rather than an “exclusion criteria”, “positive criteria” have been adopted to diagnose MAFLD, which are based on evidence of hepatic steatosis by imaging techniques, blood biomarkers, or liver histology, in addition to one of the following 3 criteria: 1) overweight/obesity, 2) type 2 diabetes mellitus, or 3) two of the seven cardiometabolic risk factors, which largely represent the criteria for metabolic syndrome (MetS) (3). The prevalence of MAFLD is expected to be more prominent because a less rigorous criterion is employed, where alcohol consumption and other concomitant liver diseases are no longer exclusion conditions, and more sensitive and inexpensive imaging techniques have come into clinical practice. The goal of the revised nomenclature is to draw public attention to the prevention and intervention of treatable metabolic dysfunction. Since specific pharmacotherapy for fatty liver disease is lacking, detection of metabolic derangement at an earlier stage to justify immediate intervention through lifestyle change should be both efficient and economical.

FibroScan is a non-invasive medical device originally designed to perform liver stiffness measurement (LSM) by vibration-controlled transient elastography (VCTE). Since 2010, controlled attenuation parameter (CAP), which is a measurement of the ultrasound attenuation coefficient, can also be measured with FibroScan devices concomitantly to LSM and has been shown to be correlated with hepatic steatosis. When taking liver biopsy as the reference, an area under the receiver operating characteristic curve (AUROC) of CAP was equal to 0.79–0.91 and 0.77–0.95 for detection of more than 5%–10% and 33% of hepatic steatosis, respectively (4–6), the performance of which is superior to that of routine ultrasonography (4, 7). Recent studies have shown a close relationship between CAP and MetS components (8–10). However, current guidance still resists routine screening for NAFLD even in high-risk subjects attending primary care, diabetes, or obesity clinics due to uncertainties related to long-term benefits and cost-effectiveness (11). Thus, further evidence is needed to justify the early screening for fatty liver disease out of consideration for detecting metabolic derangement, rather than for liver lesion sequelae alone, such as steatohepatitis, hepatic cirrhosis, or liver cancer.

In this study, a total of 922 medical staff from our hospital were recruited to investigate the association between CAP and MetS components. We aim to validate the usefulness of CAP as a surrogate marker for evaluating metabolic derangement in subjects with fewer risks, or even with no MetS components.

## Subjects/Materials and Methods

### Subjects

The medical staff in our hospital who volunteered for the NAFLD study using FibroScan (502 Touch, Echosens, France) were recruited at the time of the annual physical checkup between January and March 2018. Information about the study participants was collected including previous medical history, drinking, and smoking status. Those with chronic liver diseases, including viral hepatitis B or C, autoimmune liver disease, recent infections, prolonged use of steroids, or estrogens were excluded. Individuals with daily alcohol consumption >20 g (for males) or >10 g (for females) were not included. Subjects with alanine transaminase (ALT) or aspartate transaminase (AST) higher than 5 times or plasma creatinine level higher than 2 times the upper normal limit were also excluded from the analysis. The study was conducted in accordance with the Declaration of Helsinki and was approved by the ethics committee of the First Affiliated Hospital of Sun Yat-sen University [Ethics Committee Review (2016) No. 146]. Informed consent was obtained from each participant.

### Procedures

All subjects received clinical and laboratory assessments on the same day when the FibroScan examination was done. Anthropometric data including body weight and height, blood pressure, circumferences of the neck, waist, and hip were collected. Neck circumference was measured in the midway of the neck, between the mid-cervical spine and mid-anterior neck. Waist circumference was measured at the midpoint between the lower margin of the last palpable rib and the top of the ileal crest. Hip circumference measurement was taken around the widest portion of the buttock.

After a 10-h overnight fast, blood samples were drawn for laboratory testing, including fasting plasma glucose, lipid profiles, liver and renal function, uric acid, serum insulin, adiponectin, and whole blood cell counts. The estimated glomerular filtration rate (eGFR) was estimated using a modification of diet in renal disease (MDRD) equation for the evaluation of renal function. Indices for insulin resistance assessment included homeostatic model assessment of insulin resistance (HOMA-IR), the product of fasting triglycerides and glucose (TyG), and metabolic score for insulin resistance (METS-IR). HOMA-IR was estimated by the formula as fasting insulin (U/ml) × fasting glucose (mmol/L)/22.5. TyG was calculated as Ln [fasting triglycerides (mmol/L) × 88.6 × fasting glucose (mmol/L) × 18]/2 (12). METS-IR was defined as Ln [2 × fasting glucose (mmol/L) × 18 + fasting triglycerides (mmol/L) × 88.6) × BMI]/Ln [fasting HDL-c (mmol/L) × 38.66] (13).

All FibroScan examinations were performed by the same trained operator following the manufacturer’s instruction. CAP and LSM were assessed with the tip of the M probe placed on the skin between the ribs over the right lobe of the liver through the intercostal space. CAP was calculated only when LSM was valid to ensure an accurate attenuation value of the liver, and an attempt was made to collect ≥10 valid LSMs. The ratio of the interquartile range (IQR) to the median of the liver stiffness (IQR/Median, LSM) ≤30% was considered a reliable measurement. In our preliminary study, ROC curve analysis was performed to determine the cutoff value of CAP for diagnosing MetS. According to the maximum Youden’s index, CAP ≥ 245 dB/m was used as a cutoff point in our group, with 79.6% sensitivity and 61.6% specificity. The AUROC was 0.706 (0.665, 0.747), which indicated a moderate efficiency. In contrast, when using CAP ≥ 238 dB/m, which was used as the cutoff point for hepatic steatosis previously, the sensitivity was 82.3%, the specificity was 55.7%, and the AUROC was 0.690 (0.649, 0.731). The diagnostic power of both was similar, while the latter had slightly higher sensitivity in diagnosing MetS in our group (**Supplementary Figure 1**). Thus, hepatic steatosis grade was decided by the cutoff values of CAP according to previous reports, where CAP < 238 dB/m denoted no steatosis (S0), 238 ≤ CAP ≤ 259 dB/m denoted mild (S1), 260 ≤ CAP ≤ 291 dB/m denoted moderate (S2), and CAP > 291 dB/m denoted severe steatosis (S3) (4, 10). The hepatic fibrosis cutoff value was LSM ≥ 7.0 kPa for significant fibrosis (10, 14) (**Supplementary Figure 2**).

According to the guideline developed by the Chinese Diabetes Society in 2017, MetS was diagnosed in subjects with more than any 3 of the following 5 components: 1) waist circumference ≥90 cm in men and ≥85 cm in women; 2) blood pressure ≥130/85 mmHg or treated with anti-hypertensive drugs; 3) fasting plasma glucose ≥6.1 mmol/L, 2-h post-load glucose ≥7.8 mmol/L, or treated with anti-hyperglycemic drugs; 4) fasting triglyceride ≥1.70 mmol/L; and 5) fasting high-density lipoprotein cholesterol (HDL-c) <1.04 mmol/L. Overweight was defined as body mass index (BMI) ≥ 24 kg/m^2^, whereas obesity was BMI ≥ 28 kg/m^2^ for the Chinese population (15).

### Statistical Analysis

The Kolmogorov–Smirnov test was used to examine the distribution of all the continuous quantitative variables before comparison. Continuous variables with normal distribution were expressed as mean ± SD, or median (Q1, Q3) when normal distribution was not achieved. Categorical variables were expressed as counts and percentages. Continuous variables were compared using Student’s *t*-test or Mann–Whitney test between 2 groups, or one-way ANOVA or Kruskal–Wallis test for more than two groups’ comparison as appropriate. Bonferroni correction test was applied for *post-hoc* pairwise comparisons. Categorical variables were compared using the chi-squared test, and the 2×C cross-tabulation test was used for multiple comparisons, with Bonferroni-adjusted Z-tests for column proportions. Binary logistic regression analysis was used to calculate the odds ratios for the presence of MetS in different CAP-based hepatic steatosis groups, or in BMI and CAP combination categories, and for hepatic steatosis (CAP ≥ 238 dB/m) in subjects with no MetS components. All statistical analyses were performed using SPSS version 23.0. All statistical tests were two-sided and were evaluated at the 0.05 level of significance.

## Results

**Figure 1:**
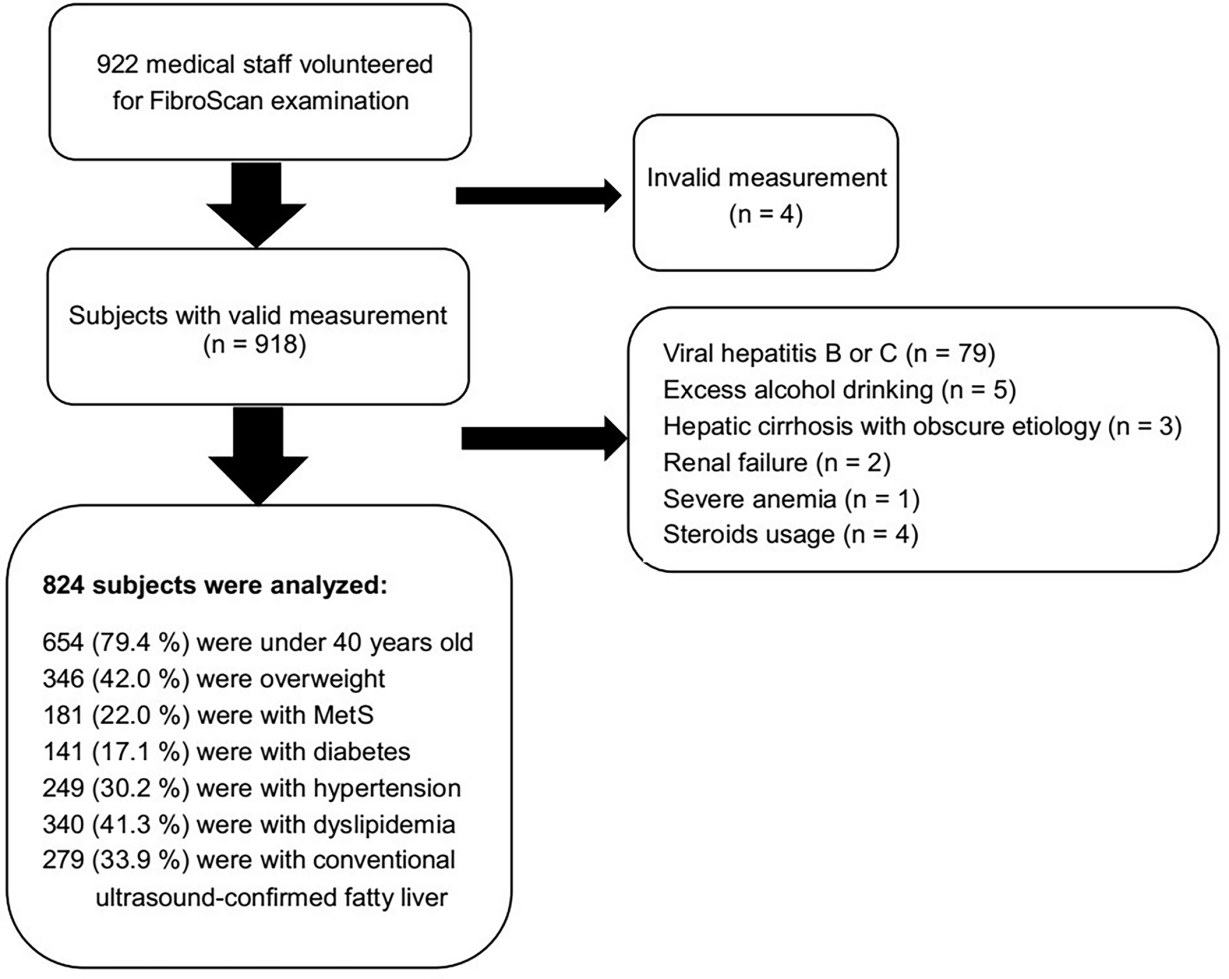
Flow chart of inclusion/exclusion and composition of the included subjects.

A total of 922 medical staff volunteered for the FibroScan examination, while 98 subjects were excluded (**Figure 1**). Data of anthropometries and laboratory tests of 824 participants were included for analysis. The average age of the included subjects was 53.2 years, and 24.4% were male subjects. The mean BMI was 23.6 kg/m^2^, while 42.0% of them were overweight, and 7.9% were obese. One hundred forty-one (17.1%) subjects were diagnosed with diabetes. Three hundred forty (41.3%) subjects had a previous history of dyslipidemia, 120 of them were treated with statins, and 15 subjects were treated with fibrates. One hundred eighty-one (22.0%) subjects met the criteria for MetS. Subjects in the MetS group were approximately 13 years older, had a slightly more male subject composition, and had significantly higher BMI, waist and neck circumferences, and waist-to-hip ratio. The median systolic and diastolic pressure was more than 10 mmHg higher in the MetS group. Around one-third of the subjects were diagnosed with fatty liver by using conventional ultrasound imaging, the percentage of which was significantly lower than that identified by FibroScan (52.7%) using CAP ≥ 238 dB/m as a cutoff value. The major difference was attributable to the fact that FibroScan detected more fatty liver in the non-MetS group than conventional ultrasonography, while the performances of both in the MetS group were similar (285/149 vs. 146/133, *p* = 0.000). The median LSM of all subjects was 4.0 kPa, only 18 subjects (2.2%) were found to have significant fibrosis (LSM ≥ 7.0 kPa), and 13 of them were in the MetS group (13/181 vs. 5/643, *p* = 0.000). The subjects in the MetS group had significantly higher fasting plasma glucose, serum insulin, triglyceride, uric acid, creatinine, ALT, AST level, and white blood cell count; their HDL-c, adiponectin, and eGFR were lower; and their total cholesterol and low-density lipoprotein cholesterol (LDL-c) were comparable with those of the non-MetS group. The CAP and LSM values, as well as insulin resistance indices including TyG, METS-IR, and HOMA-IR, were all significantly higher in the MetS group (**Table 1**).

**Table 1:** Clinical characteristics of the study population and comparisons between subjects with and without MetS.

Characteristics	Total	Non-MetS	MetS	*p*
	(n = 824)	(n = 643, 78%)	(n = 181, 22%)	
Age (years)	53.2 ± 15.4	50.4 ± 15.0	63.3 ± 12.6	0.000
Gender, male, n (%)	201 (24.4%)	146 (22.7%)	55 (30.4%)	0.034
Body mass index (kg/m^2^)	23.6 ± 3.1	23.0 ± 2.7	25.9 ± 3.1	0.000
Waist circumference (cm)	81.7 ± 9.0	79.4 ± 8.1	89.5 ± 7.2	0.000
Waist-to-hip ratio	0.87 ± 0.06	0.86 ± 0.06	0.92 ± 0.05	0.000
Neck circumference (cm)	34.0 (32.4, 37.0)	33.5 (32.0, 36.0)	36.5 (34.5, 40.1)	0.000
Systolic pressure (mmHg)	121 (111, 135)	120 (110, 130)	136 (126, 147)	0.000
Diastolic pressure (mmHg)	72 (67, 80)	70 (65, 78)	80 (74, 85)	0.000
Drinking status, n (%)	139 (16.9%)	116 (18.0%)	23 (12.7%)	0.091
Smoking status, n (%)	49 (5.9%)	32 (5.0%)	17 (9.4%)	0.026
Diabetes, n (%)	141 (17.1%)	46 (7.2%)	95 (52.5%)	0.000
Hypertension, n (%)	249 (30.2%)	123 (19.1%)	126 (69.6%)	0.000
Dyslipidemia, n (%)	340 (41.3%)	206 (32.0%)	134 (74.0%)	0.000
Stroke, n (%)	15 (1.8%)	8 (1.2%)	7 (3.9%)	0.020
Coronary arterial disease, n (%)	68 (8.3%)	38 (5.9%)	30 (16.6%)	0.000
Fatty liver by ultrasound, n (%)	279 (33.9%)	146 (22.7%)	133 (73.5%)	0.000
Controlled attenuation parameter (dB/m)	241 (216, 278)	232 (207, 261)	285 (249, 318)	0.000
CAP ≥ 238 dB/m, n (%)	434 (52.7%)	285 (44.3%)	149 (82.3%)	0.000
Liver stiffness measurement (kPa)	4.0 (3.5, 4.8)	3.9 (3.4, 4.5)	4.5 (3.8, 5.9)	0.000
Fasting glucose (mmol/L)	5.2 (4.8, 5.9)	5.1 (4.8, 5.6)	6.4 (5.7, 7.8)	0.000
Cholesterol (mmol/L)	5.2 (4.6, 5.9)	5.1 (4.6, 5.8)	5.4 (4.5, 6.2)	0.259
Triglyceride (mmol/L)	1.14 (0.85, 1.67)	1.06 (0.77, 1.49)	1.85 (1.19, 2.66)	0.000
LDL-c (mmol/L)	3.40 ± 0.81	3.41 ± 0.75	3.37 ± 0.95	0.621
HDL-c (mmol/L)	1.44 ± 0.35	1.50 ± 0.35	1.27 ± 0.31	0.000
Alanine transaminase (U/L)	18 (14, 26)	17 (13, 24)	22 (17, 30)	0.000
Aspartate transaminase (U/L)	22 (18, 26)	22 (18, 25)	23 (19, 27)	0.008
Creatinine (μmol/L)	62 (55, 73)	62 (55, 72)	67 (56, 79)	0.005
Estimated glomerular filtration rate (ml/min/1.73 m^2^)	93.5 ± 21.3	95.1 ± 20.0	88.0 ± 24.5	0.001
Uric acid (μmol/L)	335 (280, 401)	325 (274, 384)	382 (324, 448)	0.000
Adiponectin (μg/ml)	12.7 (7.7, 22.6)	13.7 (8.7, 23.5)	7.9 (4.7, 13.5)	0.000
Fasting serum insulin (μU/ml)	5.7 (3.9, 8.0)	5.2 (3.6, 7.3)	8.7 (6.7, 11.8)	0.000
White blood cell count (×10^9^/L)	6.3 (5.3, 7.4)	6.2 (5.2, 7.3)	6.8 (5.9, 7.9)	0.000
TyG	4.61 (4.41, 4.82)	4.55 (4.37, 4.73)	4.92 (4.72, 5.14)	0.000
METS-IR	31.9 (27.8, 36.8)	30.2 (26.8, 33.7)	37.7 (34.2, 42.9)	0.000
HOMA-IR	1.33 (0.87, 1.97)	1.14 (0.81, 1.71)	2.51 (1.71, 3.47)	0.000

MetS, metabolic syndrome; HOMA-IR, homeostatic model assessment of insulin resistance; TyG, the product of fasting triglycerides and glucose; METS-IR, metabolic score for insulin resistance; HDL-c, high-density lipoprotein cholesterol; LDL-c, low-density lipoprotein cholesterol.

**Figure 2:**
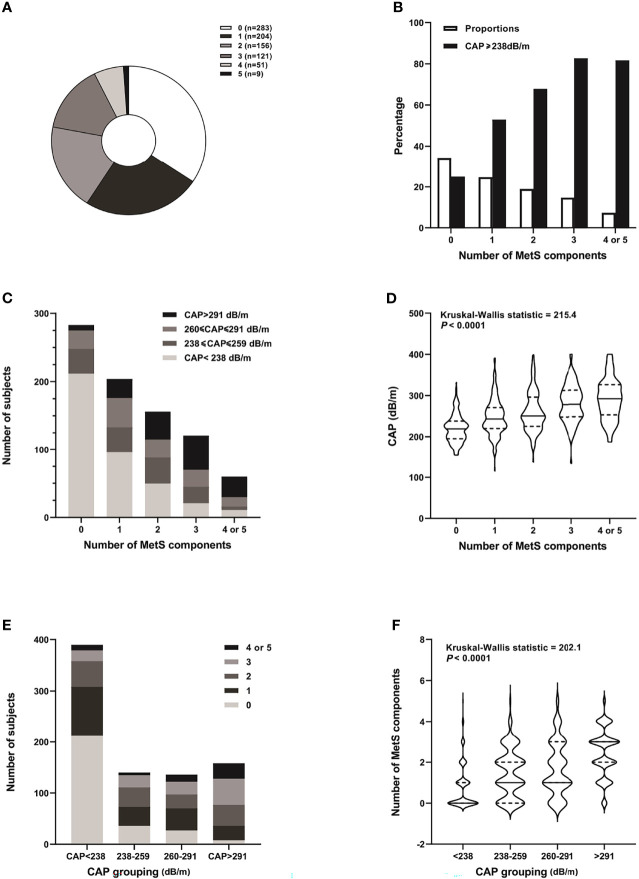
Distribution of MetS components and CAP grades of the study subjects. **(A)** Pie chart distribution of the number of MetS components. **(B)** Proportion of subjects with different number of MetS components and percentage of those with CAP ≥ 238 db/m in each group. **(C)** Number of subjects with different grades of hepatic steatosis according to CAP in each MetS component group. **(D)** Violin chart distribution of the median and quartiles of CAP in each MetS component group. **(E)** Number of subjects with 0-5 MetS components in different hepatic steatosis groups according to CAP. **(F)** Violin chart distribution of the median and quartiles of number of MetS components in different CAP-based groups.

The number of subjects with 5 MetS components was small (n = 9), so we combined them with subjects with 4 MetS components (n = 51) for further analysis. With accumulating number of MetS components, there was an upward trend in the percentage of subjects with CAP ≥ 238 dB/m in each group, from 25.1% in those with no MetS components to 81.7% in subjects with 4 or 5 MetS components. The number and percentage of subjects with CAP < 238 dB/m and 238 ≤ CAP ≤ 259 dB/m decreased, while those with CAP > 291 dB/m increased with accumulating MetS components. Likewise, the median and quartiles of CAP values increased with accumulating MetS components. When the subjects were categorized into 4 different groups according to hepatic steatosis severity as assessed by CAP measurement, the number and percentage of subjects with more MetS components (with 3, 4, or 5) increased, while those with no MetS components decreased with the increment of CAP-based groups. Similarly, the median and quartiles of the number of MetS components were also increased with increasing CAP-based groups (**Figure 2**).

There were 390 (47.3%) subjects who were diagnosed with no hepatic steatosis according to CAP, while the rest were evenly distributed among the mild, moderate, and severe hepatic steatosis groups (17.0%, 16.5%, and 19.2%, respectively) (**Table 2**). As compared with subjects with CAP < 238 dB/m, there was a notable escalating trend in age, BMI, waist and neck circumferences, waist-to-hip ratio, and blood pressure across groups with increasing CAP values. Fasting plasma glucose, serum insulin, triglyceride, ALT, AST, uric acid, white blood cell count, and indices of insulin resistance (TyG, METS-IR, and HOMA-IR) also increased, while adiponectin, HDL-c, and eGFR decreased accordingly. The differences between adjacent groups in cholesterol, LDL-c, AST, eGFR, white blood cell count, and LSM were not significant. Bonferroni *post-hoc* analysis showed that most of the clinical features in subjects with CAP < 238 dB/m were significantly different from those with CAP ≥ 238 dB/m, while differences between mild and moderate, or moderate and severe hepatic steatosis were less prominent (**Supplementary Table 1**). Among the 390 subjects with CAP < 238 dB/m, 32 (8.2%) of them met the criteria for MetS, while more than half of the subjects (51.3%) with CAP > 291 dB/m were categorized as having 3 or more MetS components. Taking the group with no hepatic steatosis (CAP < 238 dB/m) as control, subjects with mild, moderate, and severe hepatic steatosis had increased odds of having MetS by 2.92-, 4.50-, and 11.8-fold, respectively (*p* = 0.000). The odds were still significant after adjusting for multiple confounders (*p* = 0.020) (**Table 3**).

**Table 2:** Comparison of clinical characteristics among subjects in different CAP categories.

Characteristics	Controlled attenuation parameter (dB/m)				*p*	*p*	*p*
	<238 (Group 1)	238–259 (Group 2)	260–291 (Group 3)	>291 (Group 4)	Group 1 vs. 2	Group 2 vs. 3	Group 3 vs. 4
n (%)	390 (47.3%)	140 (17.0%)	136 (16.5%)	158 (19.2%)	—	—	—
Age (years)	49.6 ± 15.5	54.0 ± 14.7	58.2 ± 14.7	57.4 ± 14.0	0.018	0.115	1.000
Gender, male, n (%)	74 (19.0%)	43 (30.7%)	38 (23.3%)	46 (29.1%)	0.004	0.613	0.824
Body mass index (kg/m^2^)	22.3 ± 2.7	23.8 ± 2.8	24.7 ± 2.8	25.7 ± 2.8	0.000	0.037	0.009
Waist circumference (cm)	77.4 ± 7.9	82.5 ± 8.0	84.8 ± 7.5	88.6 ± 7.8	0.000	0.098	0.000
Waist-to-hip ratio	0.85 ± 0.06	0.88 ± 0.06	0.89 ± 0.05	0.91 ± 0.05	0.000	0.602	0.015
Neck circumference (cm)	33.0 (31.5, 34.8)	34.5 (33.0, 37.9)	35.0 (33.4, 37.9)	36.4 (34.5, 39.3)	0.000	1.000	0.061
Systolic pressure (mmHg)	118 (110, 130)	125 (112, 140)	122 (113, 136)	130 (120, 142)	0.000	1.000	0.024
Diastolic pressure (mmHg)	70 (65, 78)	75 (68, 80)	75 (70, 80)	78 (70, 85)	0.002	1.000	0.047
Controlled attenuation parameter (dB/m)	214 (195, 225)	248 (244, 253)	272 (266, 281)	318 (302, 338)	0.000	0.000	0.000
Liver stiffness measurement (kPa)	3.9 (3.4, 4.5)	4.0 (3.5, 4.7)	4.2 (3.5, 5.0)	4.4 (3.9, 5.3)	0.746	1.000	0.112
Fasting glucose (mmol/L)	5.0 (4.7, 5.5)	5.2 (4.8, 5.8)	5.4 (5.0, 5.9)	5.8 (5.2, 6.8)	0.136	0.191	0.021
Cholesterol (mmol/L)	5.00 (4.50, 5.70)	5.10 (4.50, 6.10)	5.30 (4.60, 6.10)	5.45 (4.65, 6.10)	0.319	1.000	1.000
Triglyceride (mmol/L)	0.96 (0.72, 1.29)	1.21 (0.94, 1.75)	1.48 (0.98, 1.92)	1.69 (1.14, 2.52)	0.000	0.471	0.026
LDL-c (mmol/L)	3.30 ± 0.76	3.47 ± 0.87	3.45 ± 0.75	3.51 ± 0.88	0.355	1.000	1.000
HDL-c (mmol/L)	1.56 ± 0.37	1.41 ± 0.34	1.41 ± 0.33	1.27 ± 0.27	0.001	1.000	0.006
Alanine transaminase (U/L)	16 (12, 22)	18 (13, 23)	21 (16, 29)	24 (18, 34)	0.181	0.006	0.263
Aspartate transaminase (U/L)	20 (18, 24)	22 (18, 26)	23 (20, 27)	24 (20, 28)	0.927	0.243	1.000
Creatinine (μmol/L)	60 (54, 71)	64 (56, 77)	67 (58, 78)	62 (54, 71)	0.029	1.000	0.152
Estimated glomerular filtration rate (ml/min/1.73 m^2^)	96.2 ± 21.5	91.4 ± 19.1	87.5 ± 19.3	94.0 ± 23.1	0.146	0.765	0.056
Uric acid (μmol/L)	303 (261, 366)	341 (296, 399)	363 (308, 422)	376 (321, 446)	0.001	0.854	1.000
Adiponectin (μg/ml)	16.5 (10.7, 26.2)	11.3 (7.7, 19.4)	9.4 (5.8, 16.7)	7.8 (4.7, 13.7)	0.004	0.792	0.531
Fasting serum insulin (μU/ml)	4.45 (3.24, 6.21)	6.09 (3.95, 7.24)	6.89 (4.88, 8.88)	8.47 (6.32, 11.1)	0.008	0.082	0.021
White blood cell count (×10^9^/L)	6.0 (5.1, 7.0)	6.3 (5.6, 7.5)	6.4 (5.5, 7.6)	6.9 (6.0, 7.9)	0.063	1.000	0.146
TyG	4.48 (4.32, 4.67)	4.68 (4.43, 4.82)	4.73 (4.52, 4.93)	4.83 (4.65, 5.04)	0.000	0.224	0.010
METS-IR	28.8 (25.8, 32.4)	32.3 (29.3, 36.8)	34.1 (30.1, 38.2)	36.9 (32.9, 41.1)	0.000	0.428	0.002
HOMA-IR	1.00 (0.69, 1.46)	1.34 (0.89, 1.88)	1.72 (1.13, 2.20)	2.29 (1.56, 3.17)	0.025	0.025	0.009

CAP, controlled attenuation parameter; LSM, liver stiffness measurement; eGFR, estimated glomerular filtration rate; WBC, whit blood cell; HOMA-IR, homeostatic model assessment for insulin resistance; TyG, the product of fasting triglycerides and glucose; METS-IR, metabolic score for insulin resistance.

**Table 3:** Proportion and odds ratio of having MetS according to CAP-based categories.

CAP categories	n (%)	Model 1*	Model 2**	Model 3***
		OR (95% CI)	OR (95% CI)	OR (95% CI)
<238 dB/m	32/390 (8.2%)	1	1	1
238–259 dB/m	29/140 (20.7%)	2.92 (1.69, 5.05)	1.96 (1.05, 3.67)	3.51 (1.17, 10.6)
260–291 dB/m	39/136 (28.7%)	4.50 (2.68, 7.6)	2.00 (1.10, 3.64)	3.32 (1.17, 9.46)
>291 dB/m	81/158 (51.3%)	11.8 (7.30, 19.0)	4.81 (2.75, 8.41)	5.12 (1.83, 14.3)
*p* for trend	0.000	0.000	0.000	0.020

MetS, metabolic syndrome; CAP, controlled attenuation parameter; BMI, body mass index; UA, uric acid; WBC, white blood cell; TyG, the product of fasting triglycerides and glucose.

*Model 1: unadjusted.

**Model 2: adjusted for age, BMI, and waist.

***Model 3: adjusted for age, BMI, adiponectin, ADP, UA, WBC, and TyG.

The subjects were divided into 4 groups based on whether they were overweight or not using BMI ≥ 24 kg/m^2^ as the cutoff point, and whether they were with (CAP ≥ 238 dB/m) or without (CAP < 238 dB/m) hepatic steatosis (**Table 4**). In the same BMI category of either normal weight or overweight, subjects with CAP ≥ 238 dB/m had significantly higher BMI, waist and neck circumferences, waist-to-hip ratio, blood pressure, fasting glucose and lipid profiles, liver enzymes, white blood cell count, and insulin resistance indices, but lower adiponectin and HDL-c levels as compared with those of their counterparts. Overweight subjects with CAP ≥ 238 dB/m had the worst metabolic profiles as compared with those of the other 3 groups. Forty-six percent of the subjects in this group met the criteria for MetS, nearly 10 times that of subjects in the normal-weight and CAP < 238 dB/m group (4.9%). Interestingly, other than somatotype indices (BMI, waist circumference, and neck circumference), metabolic profiles in subjects who were normal weight but with CAP ≥ 238 dB/m were comparable with those of subjects who were overweight but with CAP < 238 dB/m, including indices of insulin resistance, such as TyG and HOMA-IR. The difference in METS-IR between these 2 groups might be due to different BMIs used in the formula. The proportions of MetS in both groups were also similar (16.8% vs. 19.8%, *p* = 0.551) (**Supplementary Table 2**). Taking the normal-weight and CAP < 238 dB/m group as control, subjects who were normal weight but with CAP ≥ 238 dB/m had 3.88 times higher odds of having MetS, while the odds in subjects who were overweight with CAP < 238 dB/m was 4.75 times higher. The odds ratio increased to 16.40 in overweight subjects with CAP ≥ 238 dB/m. After age, BMI, waist, and multiple metabolic indices were adjusted, the odds ratios in normal weight with CAP ≥ 238 dB/m and overweight with CAP < 238 dB/m were similar to those of the control. However, overweight subjects with CAP ≥ 238 dB/m still had 3.24 times higher odds of having MetS. (*p* = 0.018) (**Table 5**).

**Table 4:** Clinical characteristics in normal-weight versus overweight subjects with or without hepatic steatosis.

	Normal-weight and	Normal-weight and	Overweight and	Overweight and	*p*	*p*	*p*
Characteristics	CAP < 238 dB/m	CAP ≥ 238 dB/m	CAP < 238 dB/m	CAP ≥ 238 dB/m	Group	Group	Group
	(Group 1)	(Group 2)	(Group 3)	(Group 4)	1 vs. 2	2 vs. 3	3 vs. 4
n (%)	304 (36.9%)	173 (21.0%)	86 (10.0%)	261 (31.7%)	—	—	—
Age (years)	48.1 ± 15.1	56.9 ± 14.4	54.9 ± 15.6	56.4 ± 14.7	0.000	1.000	1.000
Gender, male, n (%)	47 (15.5%)	32 (18.5%)	27 (31.4%)	95 (36.4%)	0.391	0.020	0.399
Body mass index (kg/m^2^)	21.3 ± 1.7	22.1 ± 1.5	25.9 ± 2.5	26.6 ± 2.1	0.000	0.000	0.024
Waist circumference (cm)	75.1 ± 6.4	79.1 ± 5.7	85.9 ± 6.7	89.7 ± 6.7	0.000	0.000	0.000
Waist-to-hip ratio	0.83 ± 0.05	0.87 ± 0.05	0.88 ± 0.05	0.91 ± 0.06	0.000	0.391	0.001
Neck circumference (cm)	32.5 (31.2, 34.0)	33.5 (32.0, 34.8)	35.5 (34.0, 38.0)	37.0 (34.9, 40.0)	0.008	0.000	0.107
Systolic pressure (mmHg)	115 (109, 126)	122 (110, 139)	125 (118, 135)	128 (118, 140)	0.000	1.000	0.951
Diastolic pressure (mmHg)	70 (64, 77)	72 (67, 80)	72 (66, 80)	76 (70, 83)	0.005	1.000	0.011
Controlled attenuation parameter (dB/m)	208 (192, 224)	261 (248, 285)	221 (206, 229)	288 (261, 315)	0.000	0.000	0.000
Liver stiffness measurement (kPa)	3.9 (3.4, 4.5)	4.0 (3.4, 4.6)	3.8 (3.4, 4.5)	4.3 (3.6, 5.2)	1.000	0.771	0.000
Fasting glucose (mmol/L)	5.0 (4.7, 5.5)	5.4 (4.9, 5.9)	5.1 (4.8, 5.8)	5.6 (5.1, 6.4)	0.000	0.948	0.002
Cholesterol (mmol/L)	5.00 (4.40, 5.70)	5.40 (4.70, 6.10)	5.10 (4.70, 5.60)	5.30 (4.50, 6.05)	0.005	0.282	1.000
Triglyceride (mmol/L)	0.91 (0.68, 1.14)	1.27 (0.96, 1.82)	1.21 (0.97, 1.51)	1.55 (1.08, 2.18)	0.000	1.000	0.005
HDL-c (mmol/L)	1.60 ± 0.36	1.47 ± 0.34	1.43 ± 0.35	1.28 ± 0.27	0.003	1.000	0.008
LDL-c (mmol/L)	3.31 ± 0.80	3.47 ± 0.74	3.27 ± 0.62	3.49 ± 0.90	0.383	0.517	0.282
Alanine transaminase (U/L)	15 (12, 20)	19 (16, 27)	19 (13, 26)	21 (16, 26)	0.000	1.000	0.066
Aspartate transaminase (U/L)	20 (18, 24)	23 (20, 27)	22 (19, 26)	23 (19, 26)	0.000	0.340	1.000
Creatinine (μmol/L)	59 (54, 68)	62 (55, 70)	65 (55, 76)	67 (57, 81)	0.882	0.914	1.000
Estimated glomerular filtration rate (ml/min/1.73 m^2^)	97.5 ± 21.1	92.6 ± 20.0	91.4 ± 22.5	90.2 ± 21.3	0.097	1.000	1.000
Uric acid (μmol/L)	298 (254, 354)	339 (288, 386)	337 (288, 400)	376 (328, 445)	0.000	1.000	0.015
WBC (×10^9^/L)	5.8 (5.0, 6.9)	6.4 (5.6, 7.5)	6.4 (5.7, 7.6)	6.7 (5.8, 7.9)	0.001	1.000	1.000
Adiponectin (μg/ml)	18.7 (12.0, 27.1)	13.0 (7.9, 24.2)	10.8 (6.2, 16.0)	8.1 (5.7, 12.6)	0.006	0.584	0.624
Fasting insulin (μU/ml)	4.1 (3.0, 5.7)	6.0 (4.2, 7.8)	6.4 (4.5, 8.7)	7.8 (5.8, 10.0)	0.000	1.000	0.104
TyG	4.44 (4.30, 4.61)	4.67 (4.48, 4.89)	4.63 (4.46, 4.72)	4.77 (4.60, 4.99)	0.000	0.716	0.000
METS-IR	27.4 (25.0, 29.8)	30.2 (27.5, 32.8)	34.1 (31.5, 36.7)	38.0 (34.7, 41.4)	0.000	0.000	0.007
HOMA-IR	0.95 (0.66, 1.28)	1.37 (0.95, 2.02)	1.39 (0.98, 2.07)	1.90 (1.31, 2.80)	0.000	1.000	0.047
Metabolic syndrome, n (%)	15 (4.9%)	29 (16.8%)	17 (19.8%)	120 (46.0%)	0.000	0.551	0.000

CAP, controlled attenuation parameter; LSM, liver stiffness measurement; HDL-c, high-density lipoprotein cholesterol; LDL-c, low-density lipoprotein cholesterol; WBC, white blood cell; eGFR, estimated glomerular filtration rate; HOMA-IR, homeostatic model assessment for insulin resistance; TyG the product of fasting triglycerides and glucose; METS-IR, metabolic score for insulin resistance.

**Table 5:** Odds ratio of having MetS in normal-weight versus overweight subjects with or without hepatic steatosis.

	Model 1*	Model 2**	Model 3***	Model 4^$^
	OR (95% CI)	OR (95% CI)	OR (95% CI)	OR (95% CI)
Normal weight and CAP < 238 dB/m	1	1	1	1
Normal weight and CAP ≥ 238 dB/m	3.88 (2.02, 7.47)	2.18 (1.08, 4.42)	1.01 (0.27, 3.75)	1.01 (0.27, 3.77)
Overweight and CAP < 238 dB/m	4.75 (2.26, 9.97)	1.21 (0.51, 2.90)	0.42 (0.06, 2.72)	0.56 (0.09, 3.70)
Overweight and CAP ≥ 238 dB/m	16.40 (9.24, 29.09)	3.86 (1.93, 7.74)	4.80 (1.57, 14.67)	3.24 (1.03, 10.2)
*p* for trend	0.000	0.000	0.000	0.018

MetS, metabolic syndrome; CAP, controlled attenuation parameter; BMI, body mass index; UA, uric acid; TyG, the product of fasting triglycerides and glucose; LSM, liver stiffness measurement; ALT, alanine transaminase; AST, aspartate transaminase.

*Model 1 unadjusted.

**Model 2: adjusted for age, BMI, and waist.

***Model 3: adjusted for age, BMI, waist, adiponectin, UA, WBC, ALT, and TyG.

^$^Model 4: adjusted for gender, BMI, waist, waist-to-hip ratio, LSM, fasting insulin, creatinine, cholesterol, LDL-c, AST, fasting plasma glucose, and TyG.

There were 283 subjects who were with no MetS components in our study. Clinical characteristics were compared in these subjects based on whether they were with or without CAP ≥ 238 dB/m. Both groups were similar in gender composition, blood pressure, drinking and smoking status, LSM, cholesterol, LDL-c, HDL-c, liver enzymes, and fasting plasma glucose. Subjects with CAP ≥ 238 dB/m were approximately 4 years older. Even though they were with normal body build, adipose tissue distribution, and metabolic profiles, subjects with CAP ≥ 238 dB/m had higher BMI, waist and neck circumferences, waist-to-hip ratio, fasting serum insulin, uric acid, triglyceride, white blood cell count, and insulin resistance indices, whereas their adiponectin and eGFR were significantly lower (**Table 6**). Binary logistic regression analysis was used to calculate the odds of hepatic steatosis as determined by CAP ≥ 238 dB/m in these healthy subjects. After all the correlated factors identified by variance analysis were adjusted, including age, gender, BMI, neck circumference, waist-to-hip ratio, triglyceride, eGFR, uric acid, white blood cell count, adiponectin, and TyG, only waist circumference and HOMA-IR appeared to contribute to the increased odds of hepatic steatosis. For every 1-cm increase in waist circumference, the odds of having hepatic steatosis increased by 11%. And the odds increased by 1.39 times for every 1.0 increment in HOMA-IR (**Table 7**).

**Table 6:** Comparison of clinical characteristics in subjects with no MetS components based on whether they were with or without hepatic steatosis.

Characteristics	Controlled attenuation parameter (dB/m)		*p*
	<238	≥238	
n	212	71	—
Age (years)	41.2 ± 11.3	45.4 ± 13.2	0.011
Gender, male, n (%)	27 (12.7%)	15 (21.1%)	0.085
Body mass index (kg/m^2^)	21.5 ± 2.1	22.7 ± 2.2	0.000
Waist circumference (cm)	74.4 ± 6.0	78.3 ± 5.6	0.000
Waist-to-hip ratio	0.82 ± 0.05	0.85 ± 0.05	0.000
Neck circumference (cm)	32.5 (31.0, 33.8)	33.5 (32.0, 35.5)	0.001
Systolic pressure (mmHg)	110 (106, 120)	110 (107, 120)	0.484
Diastolic pressure (mmHg)	68 (62, 72)	69 (65, 72)	0.558
Drinking status, n (%)	39 (18.4%)	11 (15.5%)	0.720
Smoking status, n (%)	3 (1.4%)	1 (1.4%)	1.000
Dyslipidemia, n (%)	23 (10.8%)	14 (19.7%)	0.055
Controlled attenuation parameter (dB/m)	206 (189, 224)	259 (246, 280)	0.000
Liver stiffness measurement (kPa)	3.8 (3.3, 4.4)	3.7 (3.3, 4.4)	0.197
Fasting plasma glucose (mmol/L)	4.9 (4.6, 5.1)	4.9 (4.7, 5.3)	0.071
Cholesterol (mmol/L)	4.90 (4.40, 5.40)	5.00 (4.60, 5.60)	0.142
Triglyceride (mmol/L)	0.82 (0.65, 1.04)	0.94 (0.75, 1.22)	0.002
LDL-c (mmol/L)	3.26 ± 0.67	3.43 ± 0.64	0.122
HDL-c (mmol/L)	1.61 ± 0.29	1.56 ± 0.35	0.280
Alanine transaminase (U/L)	15 (12, 20)	17 (13, 22)	0.077
Aspartate transaminase (U/L)	20 (18, 23)	21 (18, 25)	0.328
Creatinine (μmol/L)	58 (53, 67)	60 (54, 72)	0.069
Estimated glomerular filtration rate (ml/min/1.73 m^2^)	102.3 ± 19.0	96.7 ± 16.5	0.031
Uric acid (μmol/L)	295 (251, 346)	334 (273, 381)	0.005
Adiponectin (μg/ml)	20.6 (12.2, 29.7)	10.7 (6.8, 20.3)	0.000
Fasting serum insulin (μU/ml)	4.14 (2.96, 5.74)	5.32 (3.72, 6.61)	0.018
White blood cell count (×10^9^/L)	5.8 (5.0, 6.7)	6.3 (4.8, 7.3)	0.041
TyG	4.38 (4.25, 4.51)	4.43 (4.31, 4.59)	0.001
METS-IR	27.1 (24.8, 29.4)	28.9 (25.5, 31.4)	0.011
HOMA-IR	0.87 (0.63, 1.26)	1.09 (0.79, 1.47)	0.023

MetS, metabolic syndrome; HOMA-IR, homeostatic model assessment of insulin resistance; TyG, the product of fasting triglycerides and glucose; METS-IR, metabolic score for insulin resistance; LDL-c, low-density lipoprotein cholesterol; HDL-c, high-density lipoprotein cholesterol.

**Table 7:** Predictive factors for increasing odds of hepatic steatosis in subjects with no MetS components.

	Wald’s	Odds*	95% CI	*p*
Waist (cm)	10.5	1.11	(1.04, 1.18)	0.001
HOMA-IR	5.85	2.39	(1.18, 4.83)	0.016
Constant	15.82			0.000

MetS, metabolic syndrome; HOMA-IR, homeostatic model assessment of insulin resistance; BMI, body mass index; TyG, the product of fasting triglycerides and glucose.

*Adjusted for age, gender, BMI, neck, waist-to-hip ratio, triglyceride, glomerular filtration rate, uric acid, white blood cell count, adiponectin, and TyG.

## Discussion

In this study, clinical data of 824 healthcare workers were analyzed for detection of hepatic steatosis using FibroScan and its association with metabolic derangement. We showed that CAP value increased in parallel with the number of MetS components, while the odds of having MetS increased significantly with increasing CAP grades. In the same category of either normal weight or overweight, metabolic profiles and insulin resistance differed significantly between subjects with and without hepatic steatosis, while clinical features and presence of MetS were similar between normal-weight subjects with CAP ≥ 238 dB/m and overweight subjects with CAP < 238 dB/m. Even in subjects with no MetS components, those with CAP ≥ 238 dB/m had higher BMI, waist and neck circumferences, waist-to-hip ratio, fasting insulin, uric acid, triglyceride, white blood cell count, and indices of insulin resistance, whereas their adiponectin and eGFR were lower. Waist circumference and HOMA-IR were predictive factors for increasing odds of hepatic steatosis.

### Non-Alcoholic Fatty Liver Disease Related to Metabolic Derangement

There is growing evidence showing that NAFLD is a multisystem disease, affecting multiple extra-hepatic organs and regulatory pathways (16, 17). Meta-analysis has shown that NAFLD doubled the incidence of type 2 diabetes as well as chronic kidney disease (18, 19). Several large prospective studies also showed that NAFLD increased the risk of fatal and non-fatal CVD events, independent of established CVD risk factors (20–22). NAFLD might be associated with such complications either as a consequence of shared cardio-metabolic risk or as an example of ectopic fat accumulation leading to insulin resistance. In fact, NAFLD is not only a simple marker of cardiac/vascular and kidney damage but may also directly contribute to the development of these complications through hepatic production of lipids, atherogenic lipoproteins, pro-inflammatory and pro-oxidant molecules, induction of hepatic/peripheral insulin resistance, and glycemia dysregulation (16, 23–25). Therefore, the significance of early detection of hepatic steatosis using reliable, non-invasive, and inexpensive techniques is essential to screen for the accompanying metabolic derangement and warrant the initiation of early intervention.

In our study, the subjects included are assumed to be at lower risk of having MetS as compared with the general population, because they have more knowledge of the healthcare consequences and disease prevention. They are engaged in more health behavioral counseling including physical activity, fruit and vegetable consumption, and ideal body weight maintenance. Indeed, the proportions of drinking (16.9%) and smoking (5.9%) in our study were relatively low, as were the percentages of obesity (7.9%), MetS (22%), and significant hepatic fibrosis (2.2%). We showed that there were clear-cut differences in metabolic derangement between subjects with and without hepatic steatosis by using CAP ≥ 238 dB/m as the cutoff value. The odds of having MetS significantly elevated with increased severity of hepatic steatosis. The metabolic profiles and odds of having MetS in normal-weight subjects with CAP ≥ 238 dB/m were very similar to those of overweight subjects with CAP < 238 dB/m, indicating that the metabolic impact of fat accumulation in the liver in a non-overweight subject is comparable with that of general fat accumulation other than the liver in an overweight subject. Even in apparently healthy subjects with no MetS components, those with CAP ≥ 238 dB/m had more anthropometric and hematological indices associated with MetS and insulin resistance. Thus, CAP is a convenient and sensitive non-invasive surrogate marker for early detection of metabolic derangement in a population with lower risk.

### Controlled Attenuation Parameter Associated With Metabolic Syndrome Components

Large and prospective studies have demonstrated the accuracy of CAP in identifying hepatic steatosis (7, 26, 27). The usefulness of CAP in exploring the correlation between NAFLD and MetS components has also been validated previously. Mikolasevic et al. (10) showed that in 648 patients with one or more MetS components, in whom 67.3% met the criteria for MetS, the prevalence of NAFLD (CAP ≥ 238 dB/m, 88.3%) and advanced liver fibrosis (LSM ≥ 7.0 kPa, 16.5%) was high, and CAP and LSM correlated with the number of MetS components. Another study involved 1,983 community-based participants who underwent self-paid health examination, in which the proportion of MetS was relatively low (13.6%). It showed that CAP had a moderate prediction performance for MetS. The AUROC was 0.79 for CAP alone, and it rose to 0.85 when combined with gender, age, and BMI (8). Huh et al. (9) investigated the interplay of obesity and metabolic health on hepatic steatosis and fibrosis in 2,198 asymptomatic subjects who underwent health checkups using FibroScan. They showed that the presence of either metabolically unhealthy status or obesity was closely associated with the risk of hepatic steatosis, while significant liver fibrosis was more significantly associated with obesity rather than with unhealthy metabolic status. Our results agree with the above studies in that CAP increased in parallel with the number of MetS components. Risks of having MetS increased with the severity of hepatic steatosis as graded by CAP. Because the proportion of significant fibrosis was quite low (2.2%) in our study, we did not confirm the effect of LSM in association with obesity, metabolic disturbance, or insulin resistance. The increment in LSM appeared to be an accompanying change with CAP, and the differences in LSM were only significant in the CAP > 291 dB/m group or overweight subjects with CAP ≥ 238 dB/m as compared with other groups in our study.

There are two significant findings in our study. Firstly, we showed the distinct differences between subjects with and without hepatic steatosis using CAP ≥ 238 dB/m as a cutoff point in the same category of either normal weight or overweight. Thus, in subjects who are with normal body habitus, those with increased CAP value tend to have worse metabolic profiles and insulin sensitivity, and the biochemical indices and proportion of patients with MetS are similar to those who are overweight and with no hepatic steatosis. Subjects who are both overweight and with hepatic steatosis have the worst metabolic profiles and the greatest risk of having MetS. Therefore, CAP ≥ 238 dB/m could serve as a sensitive surrogate marker for identifying metabolic derangement in both normal-weight and overweight populations. Subjects who are normal weight with CAP ≥ 238 dB/m might represent those who have normal total fat mass but impaired adipose tissue expandability and function. In contrast, subjects who are overweight but with CAP < 238 dB/m may represent those who have better adipose tissue function, less ectopic fat storage, and better insulin sensitivity, though with increased total fat mass. A consensus has been reached on the recognition of sub-phenotypes of body weight in association with metabolic health status, namely, “normal-weight metabolically healthy”, “normal-weight metabolically unhealthy”, “metabolically healthy obesity”, and “metabolically unhealthy obesity”, though the diagnostic criteria are not consistent, and the cardiovascular and metabolic consequences of the sub-phenotypes are disputable (28–32). Because CAP correlates well with MetS components, higher values of CAP may indicate increased metabolic derangement and insulin resistance. The role of CAP in identifying normal-weight subjects with hepatic steatosis is especially important for the Asian population since the recent guideline has highlighted the fact that Asian people are particularly susceptible to lean NAFLD, partly because of body composition differences in fat and muscle, as well as genetic susceptibility (33). More importantly, evidence showed that non-obese patients achieved equivalent remission of NAFLD by a modest (3%–5%) weight reduction through lifestyle intervention (34). Thus, prospective studies are needed to further validate the usefulness of CAP as a surrogate marker for the transition of metabolic health status over time and for the prediction of cardiovascular outcomes.

Additionally, we showed that in subjects with no MetS components, the differences between those with CAP < 238 dB/m and CAP ≥ 238 dB/m were apparent. Subjects with increased CAP value tend to be older and have higher BMI, waist and neck circumferences, waist-to-hip ratio, triglyceride, uric acid, fasting insulin, white blood cell count, and insulin resistance indices, whereas lower adiponectin and eGFR. These are routine indicators associated with metabolic derangement, insulin resistance, and chronic inflammation. Our findings may suggest that in metabolically healthy subjects with increased CAP, though within normal range, subtle adverse changes in body size, visceral adiposity, lipid profile, uric acid, inflammatory index, and insulin resistance have already clustered in the direction of MetS long before its occurrence. Because this is a cross-sectional study, we could not rule out the possibility that the differences between the two groups were due to an approximately 4 years’ gap in age by chance, which resulted in aging-associated metabolic derangement and insulin resistance. However, in the subsequent binary logistic regression analysis, age was no longer a risk factor after adjusting for multiple correlated confounders. The odds of hepatic steatosis increase with elevated waist circumference and HOMA-IR, which are indicators of visceral adipose accumulation and hepatic insulin resistance. However, long-term follow-up of these subjects for their transition of metabolic health status is still needed to validate this point of view.

### Limitations

Our study has several limitations. Firstly, we do not have our own liver biopsy data to support the cutoff point of CAP for diagnosing hepatic steatosis. As a matter of fact, age-, gender-, and ethnic-specific CAP cutoff values based on liver biopsy in the general population without known hepatic diseases are lacking. We chose the one most recognized in the literature. Nevertheless, we showed that CAP ≥ 238 dB/m is sensitive enough to distinguish between different metabolic statuses. Secondly, the proportion of male subjects is small; thus, we failed to confirm the gender discrepancy in association with MetS as described previously (35, 36). Likewise, the proportion of significant fibrosis is not enough to validate its contribution to assessing metabolic risk. Therefore, we may need a larger sample size, with a more sensible distribution across gender and liver stiffness in future studies. Finally, since this is a cross-sectional study, we cannot validate the causation between hepatic steatosis and MetS. However, the metabolic derangement between subjects with or without hepatic steatosis was evident even in those with no MetS components or in subjects with normal weight. Therefore, we believe that CAP ≥ 238 dB/m did make a significant impact on a subject, even though he/she may seem apparently healthy, with no known risk factors or metabolic derangement. Rather than label this subject with a disease name, it may be more appropriate to call for attention to the underlying or impending metabolic derangement and take action (through lifestyle modification) to avoid future adverse outcomes. We need a prospective and longitudinal study to verify our findings.

### Summary

In summary, we showed that CAP is a convenient and sensitive non-invasive surrogate marker for early detection of metabolic derangement in a population with lower risk. The major benefit of detecting hepatic steatosis in apparently healthy individuals is to identify the underlying metabolic derangement, therefore allowing the initiation of early intervention through lifestyle modification. This is of particular importance for the Asian population with a substantial proportion of lean NAFLD. Prospective studies are needed to further evaluate the usefulness of CAP as a surrogate marker for the transition of metabolic health status and for the prediction of cardiovascular outcomes.

## Data Availability Statement

The original contributions presented in the study are included in the article/**Supplementary Material**. Further inquiries can be directed to the corresponding author.

## Ethics Statement

The studies involving human participants were reviewed and approved by the ethics committee of the First Affiliated Hospital of Sun Yat-sen University. The patients/participants provided their written informed consent to participate in this study.

## Author Contributions

YL conceptualized the study, supported the execution with her fund, and revised and proofread the manuscript. ZH designed the study and wrote the manuscript with input from all the authors. ZH and KN analyzed the data. KN, HC, and WD executed the study and collected data from all the participants. All authors checked the final manuscript before submission.

## Funding

This work was supported by the National Key R&D Program of China (2018YFC1314100) and National Natural Science Fund of China (82070918).

## Conflict of Interest

The authors declare that the research was conducted in the absence of any commercial or financial relationships that could be construed as a potential conflict of interest.

## Publisher’s Note

All claims expressed in this article are solely those of the authors and do not necessarily represent those of their affiliated organizations, or those of the publisher, the editors and the reviewers. Any product that may be evaluated in this article, or claim that may be made by its manufacturer, is not guaranteed or endorsed by the publisher.
