# Supplementary figures and images for: Validation of Controlled Attenuation Parameter Measured by FibroScan as a Novel Surrogate Marker for the Evaluation of Metabolic Derangement
Source: Front Endocrinol (Lausanne). 2022 Jan 31;12:739875. doi: 10.3389/fendo.2021.739875 (PMC8841525; doi:10.3389/fendo.2021.739875)

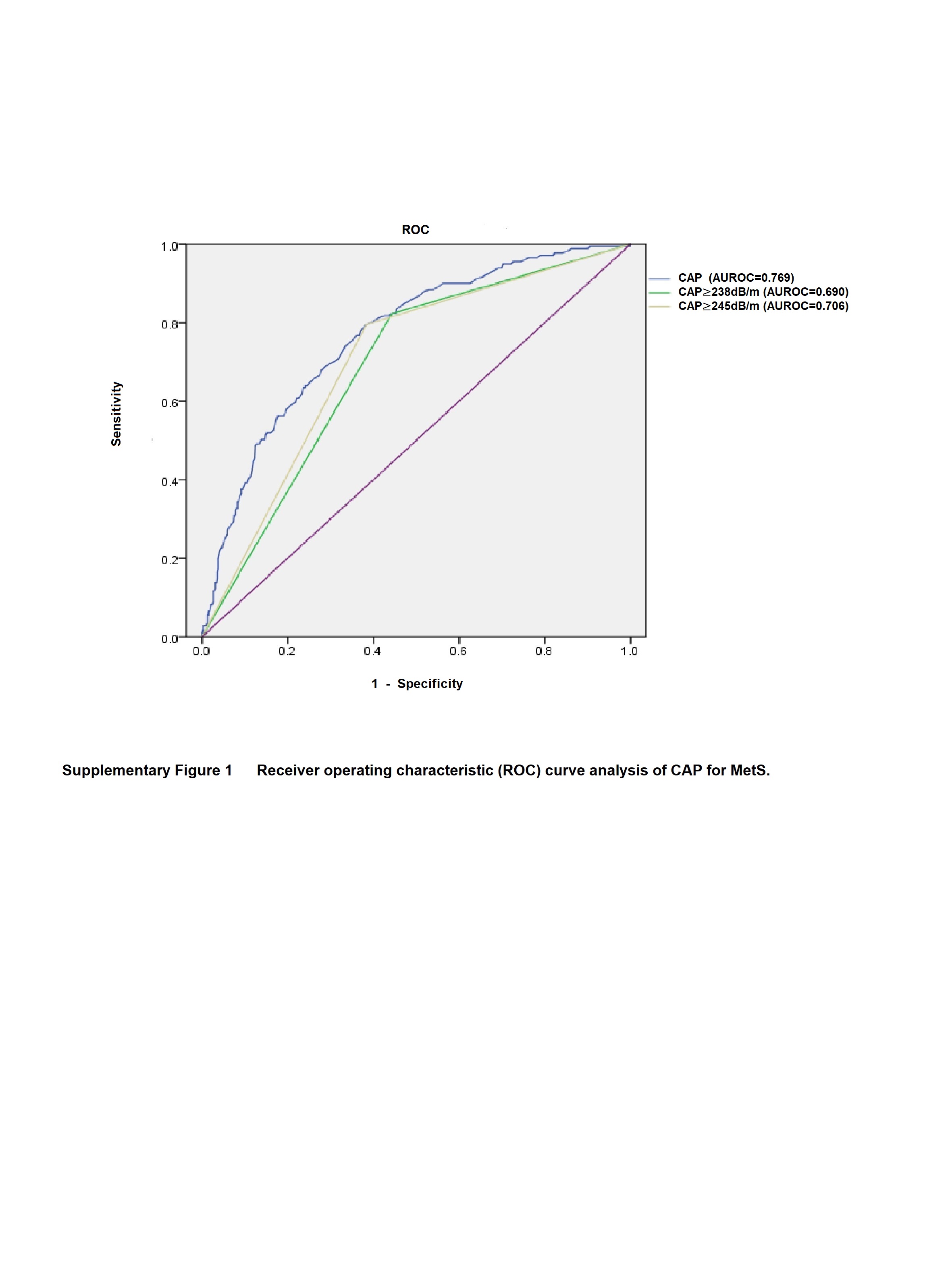

Supplement: Supplementary file 1 [file Image_1.jpeg]

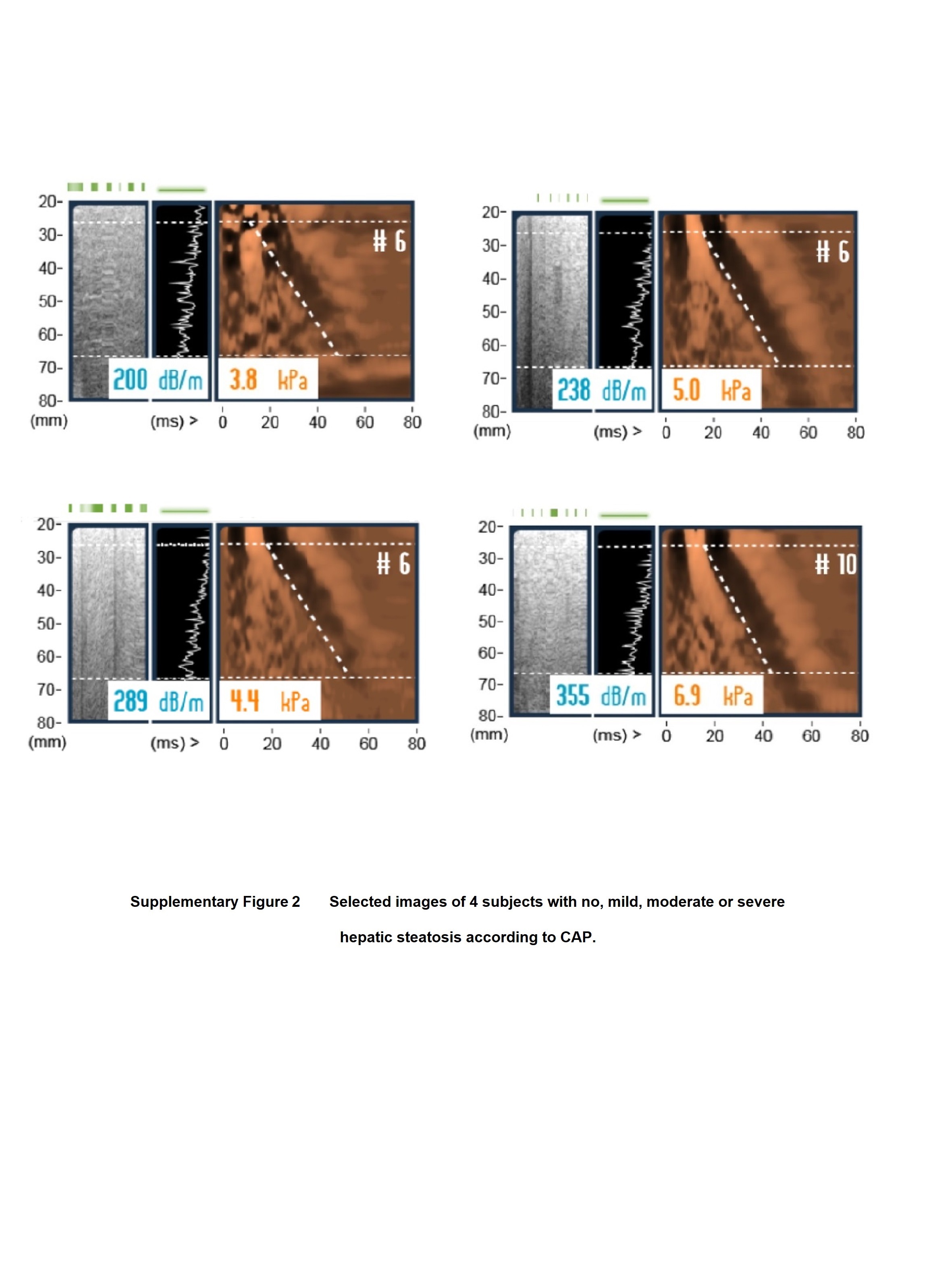

Supplement: Supplementary file 2 [file Image_2.jpeg]
