# Supplementary material for: Validation of Controlled Attenuation Parameter Measured by FibroScan as a Novel Surrogate Marker for the Evaluation of Metabolic Derangement
Source: Front Endocrinol (Lausanne). 2022 Jan 31;12:739875. doi: 10.3389/fendo.2021.739875 (PMC8841525; doi:10.3389/fendo.2021.739875)
Supplement: Supplementary file 3 [file DataSheet_1.docx]

| **Supplementary Table 1 Bonferroni post-hoc comparison between groups in different CAP categories.** | | | | | | |
| --- | --- | --- | --- | --- | --- | --- |
|  | Among groups | Between  Groups | | Adjusted *P* _1 vs 2, 3, 4_ | Adjusted  *P* _2 vs 3, 4_ | Adjusted  *P* _3 vs 4_ |
| Age (year) | 0.000 | 1 | 2 | 0.018 |  |  |
|  |  |  | 3 | 0.000 | 0.115 |  |
|  |  |  | 4 | 0.000 | 0.281 | 1.000 |
| Gender, male (%) | 0.007 | 1 | 2 | 0.004 |  |  |
|  |  |  | 3 | 0.028 | 0.613 |  |
|  |  |  | 4 | 0.009 | 0.763 | 0.824 |
| Body mass index (kg/m^2^) | 0.000 | 1 | 2 | 0.000 |  |  |
|  |  |  | 3 | 0.000 | 0.037 |  |
|  |  |  | 4 | 0.000 | 0.000 | 0.009 |
| Waist circumference (cm) | 0.000 | 1 | 2 | 0.000 |  |  |
|  |  |  | 3 | 0.000 | 0.098 |  |
|  |  |  | 4 | 0.000 | 0.000 | 0.000 |
| Waist-to-hip ratio | 0.000 | 1 | 2 | 0.000 |  |  |
|  |  |  | 3 | 0.000 | 0.602 |  |
|  |  |  | 4 | 0.000 | 0.000 | 0.015 |
| Neck circumference (cm) | 0.000 | 1 | 2 | 0.000 |  |  |
|  |  |  | 3 | 0.000 | 1.000 |  |
|  |  |  | 4 | 0.000 | 0.001 | 0.061 |
| Systolic pressure (mmHg) | 0.000 | 1 | 2 | 0.000 |  |  |
|  |  |  | 3 | 0.004 | 1.000 |  |
|  |  |  | 4 | 0.000 | 0.128 | 0.024 |
| Diastolic pressure (mmHg) | 0.000 | 1 | 2 | 0.002 |  |  |
|  |  |  | 3 | 0.003 | 1.000 |  |
|  |  |  | 4 | 0.000 | 0.053 | 0.047 |
| Controlled attenuation parameter (dB/m) | 0.000 | 1 | 2 | 0.000 |  |  |
|  |  |  | 3 | 0.000 | 0.000 |  |
|  |  |  | 4 | 0.000 | 0.000 | 0.000 |
| Liver stiffness measurement (kPa) | 0.000 | 1 | 2 | 0.746 |  |  |
|  |  |  | 3 | 0.042 | 1.000 |  |
|  |  |  | 4 | 0.000 | 0.004 | 0.112 |
| Fasting glucose (mmol/L) | 0.000 | 1 | 2 | 0.136 |  |  |
|  |  |  | 3 | 0.000 | 0.190 |  |
|  |  |  | 4 | 0.000 | 0.000 | 0.021 |
| Cholesterol (mmol/L) | 0.006 | 1 | 2 | 0.319 |  |  |
|  |  |  | 3 | 0.162 | 1.000 |  |
|  |  |  | 4 | 0.011 | 1.000 | 1.000 |
| Triglyceride (mmol/L) | 0.000 | 1 | 2 | 0.000 |  |  |
|  |  |  | 3 | 0.000 | 0.471 |  |
|  |  |  | 4 | 0.000 | 0.000 | 0.026 |
| LDL-c (mmol/L) | 0.037 | 1 | 2 | 0.355 |  |  |
|  |  |  | 3 | 0.483 | 1.000 |  |
|  |  |  | 4 | 0.063 | 1.000 | 1.000 |

|  | Among groups | Between groups | | Adjusted *P* _1 vs 2, 3, 4_ | Adjusted  *P* _2 vs 3, 4_ | Adjusted  *P* _3 vs 4_ |
| --- | --- | --- | --- | --- | --- | --- |
| HDL-c (mmol/L) | 0.000 | 1 | 2 | 0.001 |  |  |
|  |  |  | 3 | 0.000 | 1.000 |  |
|  |  |  | 4 | 0.000 | 0.005 | 0.006 |
| Alanine transaminase (U/L) | 0.000 | 1 | 2 | 0.181 |  |  |
|  |  |  | 3 | 0.000 | 0.006 |  |
|  |  |  | 4 | 0.000 | 0.000 | 0.263 |
| Aspartate transaminase (U/L) | 0.000 | 1 | 2 | 0.927 |  |  |
|  |  |  | 3 | 0.001 | 0.243 |  |
|  |  |  | 4 | 0.000 | 0.024 | 1.000 |
| Creatinine (μmol/L) | 0.001 | 1 | 2 | 0.029 |  |  |
|  |  |  | 3 | 0.001 | 1.000 |  |
|  |  |  | 4 | 1.000 | 0.824 | 0.152 |
| Estimated glomerular filtration ratio (ml/min/1.73m^2^) | 0.000 | 1 | 2 | 0.146 |  |  |
|  |  |  | 3 | 0.000 | 0.765 |  |
|  |  |  | 4 | 1.000 | 1.000 | 0.056 |
| Uric acid (μmol/L) | 0.000 | 1 | 2 | 0.001 |  |  |
|  |  |  | 3 | 0.000 | 0.854 |  |
|  |  |  | 4 | 0.000 | 0.024 | 1.000 |
| Adiponectin (μg/mL) | 0.000 | 1 | 2 | 0.004 |  |  |
|  |  |  | 3 | 0.000 | 0.792 |  |
|  |  |  | 4 | 0.000 | 0.007 | 0.531 |
| Fasting serum insulin (μU/mL) | 0.000 | 1 | 2 | 0.008 |  |  |
|  |  |  | 3 | 0.000 | 0.082 |  |
|  |  |  | 4 | 0.000 | 0.000 | 0.021 |
| White blood cell count (×10^9^/L) | 0.000 | 1 | 2 | 0.063 |  |  |
|  |  |  | 3 | 0.021 | 1.000 |  |
|  |  |  | 4 | 0.000 | 0.054 | 0.146 |
| TyG | 0.000 | 1 | 2 | 0.000 |  |  |
|  |  |  | 3 | 0.000 | 0.224 |  |
|  |  |  | 4 | 0.000 | 0.000 | 0.010 |
| METS-IR | 0.000 | 1 | 2 | 0.000 |  |  |
|  |  |  | 3 | 0.000 | 0.428 |  |
|  |  |  | 4 | 0.000 | 0.000 | 0.002 |
| HOMA-IR | 0.000 | 1 | 2 | 0.025 |  |  |
|  |  |  | 3 | 0.000 | 0.025 |  |
|  |  |  | 4 | 0.000 | 0.000 | 0.009 |

*CAP* controlled attenuation parameter, *HOMA-IR* homeostatic model assessment for insulin resistance, *TyG* the product of fasting triglycerides and glucose, *METS-IR* metabolic score for insulin resistance.

Group 1: CAP<238dB/m; Group 2: 238≤CAP≤259dB/m; Group 3: 260≤CAP≤291dB/m; Group 4: CAP>291dB/m.

| **Supplementary Table 2 Bonferroni post-hoc analysis between groups in** normal-weight versus overweight with or without hepatic steatosis. | | | | | | |
| --- | --- | --- | --- | --- | --- | --- |
|  | Among groups | Between groups | | Adjusted *P* _1 vs 2, 3, 4_ | Adjusted  *P* _2 vs 3, 4_ | Adjusted  *P* _3 vs 4_ |
| Age (year) | 0.000 | 1 | 2 | 0.000 |  |  |
|  |  |  | 3 | 0.001 | 1.000 |  |
|  |  |  | 4 | 0.000 | 1.000 | 1.000 |
| Gender, male (%) | 0.000 | 1 | 2 | 0.391 |  |  |
|  |  |  | 3 | 0.001 | 0.020 |  |
|  |  |  | 4 | 0.000 | 0.000 | 0.399 |
| Body mass index (kg/m^2^) | 0.000 | 1 | 2 | 0.000 |  |  |
|  |  |  | 3 | 0.000 | 0.000 |  |
|  |  |  | 4 | 0.000 | 0.000 | 0.024 |
| Waist circumference (cm) | 0.000 | 1 | 2 | 0.000 |  |  |
|  |  |  | 3 | 0.000 | 0.000 |  |
|  |  |  | 4 | 0.000 | 0.000 | 0.000 |
| Waist-to-hip ratio | 0.000 | 1 | 2 | 0.000 |  |  |
|  |  |  | 3 | 0.000 | 0.391 |  |
|  |  |  | 4 | 0.000 | 0.000 | 0.001 |
| Neck circumference (cm) | 0.000 | 1 | 2 | 0.008 |  |  |
|  |  |  | 3 | 0.000 | 0.000 |  |
|  |  |  | 4 | 0.000 | 0.000 | 0.107 |
| Systolic pressure (mmHg) | 0.000 | 1 | 2 | 0.000 |  |  |
|  |  |  | 3 | 0.000 | 1.000 |  |
|  |  |  | 4 | 0.000 | 0.032 | 0.951 |
| Diastolic pressure (mmHg) | 0.000 | 1 | 2 | 0.005 |  |  |
|  |  |  | 3 | 0.165 | 1.000 |  |
|  |  |  | 4 | 0.000 | 0.003 | 0.011 |
| Controlled attenuation parameter (dB/m) | 0.000 | 1 | 2 | 0.000 |  |  |
|  |  |  | 3 | 0.308 | 0.000 |  |
|  |  |  | 4 | 0.000 | 0.012 | 0.000 |
| Liver stiffness measurement (kPa) | 0.000 | 1 | 2 | 1.000 |  |  |
|  |  |  | 3 | 1.000 | 0.771 |  |
|  |  |  | 4 | 0.000 | 0.010 | 0.000 |
| Fasting glucose (mmol/L) | 0.000 | 1 | 2 | 0.000 |  |  |
|  |  |  | 3 | 0.387 | 0.948 |  |
|  |  |  | 4 | 0.000 | 0.041 | 0.002 |
| Cholesterol (mmol/L) | 0.005 | 1 | 2 | 0.005 |  |  |
|  |  |  | 3 | 1.000 | 0.282 |  |
|  |  |  | 4 | 0.097 | 1.000 | 1.000 |
| Triglyceride (mmol/L) | 0.000 | 1 | 2 | 0.000 |  |  |
|  |  |  | 3 | 0.000 | 1.000 |  |
|  |  |  | 4 | 0.000 | 0.025 | 0.005 |
| HDL-c (mmol/L) | 0.000 | 1 | 2 | 0.003 |  |  |
|  |  |  | 3 | 0.001 | 1.000 |  |
|  |  |  | 4 | 0.000 | 0.000 | 0.008 |

|  | Among groups | Between groups | | Adjusted *P* _1 vs 2, 3, 4_ | Adjusted  *P* _2 vs 3, 4_ | Adjusted  *P* _3 vs 4_ |
| --- | --- | --- | --- | --- | --- | --- |
| LDL-c (mmol/L) | 0.040 | 1 | 2 | 0.383 |  |  |
|  |  |  | 3 | 1.000 | 0.517 |  |
|  |  |  | 4 | 0.123 | 1.000 | 0.282 |
| Alanine transaminase (U/L) | 0.000 | 1 | 2 | 0.000 |  |  |
|  |  |  | 3 | 0.008 | 1.000 |  |
|  |  |  | 4 | 0.000 | 0.721 | 0.066 |
| Aspartate transaminase (U/L) | 0.000 | 1 | 2 | 0.000 |  |  |
|  |  |  | 3 | 0.403 | 0.340 |  |
|  |  |  | 4 | 0.001 | 0.700 | 1.000 |
| Creatinine (μmol/L) | 0.000 | 1 | 2 | 0.882 |  |  |
|  |  |  | 3 | 0.047 | 0.914 |  |
|  |  |  | 4 | 0.000 | 0.015 | 1.000 |
| Estimated glomerular filtration ratio (ml/min/1.73m^2^) | 0.000 | 1 | 2 | 0.097 |  |  |
|  |  |  | 3 | 0.128 | 1.000 |  |
|  |  |  | 4 | 0.000 | 1.000 | 1.000 |
| Uric acid (μmol/L) | 0.000 | 1 | 2 | 0.000 |  |  |
|  |  |  | 3 | 0.001 | 1.000 |  |
|  |  |  | 4 | 0.000 | 0.000 | 0.015 |
| White blood cell count (×10^9^/L) | 0.000 | 1 | 2 | 0.001 |  |  |
|  |  |  | 3 | 0.006 | 1.000 |  |
|  |  |  | 4 | 0.000 | 0.505 | 1.000 |
| Adiponectin (μg/mL) | 0.000 | 1 | 2 | 0.006 |  |  |
|  |  |  | 3 | 0.000 | 0.584 |  |
|  |  |  | 4 | 0.000 | 0.000 | 0.624 |
| Fasting serum insulin (μU/mL) | 0.000 | 1 | 2 | 0.000 |  |  |
|  |  |  | 3 | 0.000 | 1.000 |  |
|  |  |  | 4 | 0.000 | 0.000 | 0.104 |
| TyG | 0.000 | 1 | 2 | 0.000 |  |  |
|  |  |  | 3 | 0.000 | 0.716 |  |
|  |  |  | 4 | 0.000 | 0.020 | 0.000 |
| METS-IR | 0.000 | 1 | 2 | 0.000 |  |  |
|  |  |  | 3 | 0.000 | 0.000 |  |
|  |  |  | 4 | 0.000 | 0.000 | 0.007 |
| HOMA-IR | 0.000 | 1 | 2 | 0.000 |  |  |
|  |  |  | 3 | 0.000 | 1.000 |  |
|  |  |  | 4 | 0.000 | 0.000 | 0.047 |
| Metabolic syndrome (%) | 0.000 | 1 | 2 | 0.000 |  |  |
|  |  |  | 3 | 0.000 | 0.551 |  |
|  |  |  | 4 | 0.000 | 0.000 | 0.000 |

*CAP* controlled attenuation parameter, *HOMA-IR* homeostatic model assessment for insulin resistance, *TyG* the product of fasting triglycerides and glucose, *METS-IR* metabolic score for insulin resistance.

Group 1: BMI < 24kg/m^2^ & CAP < 238dB/m; Group 2: BMI < 24kg/m^2^ & CAP ≥ 238dB/m;

Group 3: BMI ≥ 24kg/m^2^ & CAP < 238dB/m; Group 4: BMI ≥ 24kg/m^2^ & CAP ≥ 238dB/m.
